# Knowledge of health practitioners regarding mental health integration into human immunodeficiency virus management into primary healthcare level

**DOI:** 10.4102/curationis.v43i1.2041

**Published:** 2020-08-20

**Authors:** Winnie B. Cele, Euphemia M. Mhlongo

**Affiliations:** 1 School of Nursing & Public Health, University of KwaZulu-Natal, Durban, South Africa

**Keywords:** integration, mental health, mental health care user, HIV, primary healthcare

## Abstract

**Background:**

Mental disorders are common among people living with human immunodeficiency virus (HIV). Human immunodeficiency virus infection is associated with neurological complications, which may negatively affect antiretroviral treatment, leading to virologic as well as immunologic failure. The integration of mental healthcare services into HIV services at a primary healthcare level is vital, as this is the first contact point for most patients accessing healthcare services.

**Objectives:**

The aim of this study was to determine the knowledge of healthcare professionals about the integration of mental healthcare services into HIV services at a primary healthcare level.

**Method:**

This study was a quantitative descriptive study, designed to determine the knowledge of healthcare professionals towards integrating mental healthcare services into routine services at primary healthcare centres. The purposive sampling method was employed to select the 200 healthcare professionals who participated in this study.

**Results:**

The majority of the healthcare professionals (91%) who participated in this study had limited knowledge regarding the integration of mental healthcare services into HIV services at a primary healthcare level. Very few (9%, *n* = 18) had adequate knowledge about the integration of mental healthcare services into HIV services.

**Conclusion:**

Most of the participants had limited knowledge about the subject matter. This, therefore, shows that all stakeholders involved need to implement initiatives to address this knowledge gap.

## Background of the study

Mental disorders seem to be significantly widespread among people living with human immunodeficiency virus (PLWH) which can also be corroborated by the fact that major depressive disorders (MDD) occur nearly twice as often among PLWH than the general population (Johnsson 2013). Common mental disorders in low- and middle-income countries (LMICs) occur in about 30% of PLWH (Chibanda et al. 2014). The burden of HIV is felt significantly on a global scale, with estimates showing that there are 36.9 million PLWH worldwide (Chuah et al. 2017). Research shows that in South Africa, close to 25% of PLWH are affected by one form of mental health condition or another (Modula & Ramukumba 2018).

The connection between mental health conditions and HIV remains elusive. It is, however, known that the impact of HIV infection on the immune system and the opportunistic infections that are commonly linked with AIDS may result in neurological complications (Dubé et al. 2005). Additionally, mental health challenges may develop as adverse side effects of the use of antiretroviral therapy or from other socio-economic predicaments (Chuah et al. 2017; Yi et al. 2015). Although the use of antiretroviral drugs has tremendously changed HIV infection management, decreasing morbidity and mortality, there is still a need for effective follow-up by caregivers to achieve optimal results and also to ensure adherence to medication (Carvalhal et al. 2012). Poor adherence to antiretroviral therapy is associated with psychiatric co-morbidities, and this lends support to a virologic as well as an immunologic failure (Carvalhal 2015). Co-morbidities have the potential to impede effective healthcare delivery and may cause an increase in physical and emotional strain on both formal and informal healthcare providers (Carvalhal 2015; Ciesla & Roberts 2001).

Because of limited finances, human resources and necessary healthcare infrastructures in LMICs, healthcare delivery systems are usually overstressed. They are organised in a model of handling acute conditions, excluding South Africa since their ideal clinic strategy is implemented, thus leading to fragmentation in healthcare service delivery as well as poor sustainability of healthcare for conditions that need long-term management, such as mental health issues and HIV infection (Semrau et al. 2015). These problems may be addressed by the integration of mental healthcare services into HIV services. Benefits that arise from the integration of mental healthcare services into HIV services, at a primary healthcare level, include the provision all-inclusive healthcare and the decrease in stigma that is associated with both HIV and mental health issues. Integration of mental healthcare services into HIV services will make the sharing of existing healthcare facilities possible for the management of these disorders leading to better service delivery (Petersen et al. 2016). The management of PLWH is becoming increasingly complex and requires the putting in place of innovative healthcare delivery models to address all complications that arise from HIV infection. Such models of healthcare delivery should involve multidisciplinary teams for effective collaboration (Brennan et al. 2015).

The importance of integrating mental healthcare services into HIV services at a primary healthcare level cannot be stressed enough, as it will afford healthcare professionals who are managing the patients, with the opportunity to be well skilled in the management of this co-morbid condition. This will help to resolve the under-diagnosis of mental disorders in PLWH, as untreated mental disorders substantially affect the health outcomes in PLWH (Johnsson 2013). This puts a great demand on healthcare providers to effectively diagnose and treat mental health issues in the population of PLWH (Thom 2012).

It has been reported that just a few healthcare services are in existence globally that meet the mental health needs of people infected with HIV, and thus, there is a need to change this narrative (UNAIDS 2018). Robust healthcare delivery systems that take into consideration the multifaceted medical needs of HIV-infected people, ranging from psychological to social needs, must be instituted. And this can be best achieved via the implementation of integrated programmes (UNAIDS 2018).

## Aims and objectives

The aim of this study was to determine the knowledge of healthcare professionals regarding the integration of mental healthcare services into HIV services at a primary healthcare level.

## Method

A quantitative and descriptive design was employed to determine the knowledge of healthcare professionals regarding the implementation of mental healthcare services into HIV services at a primary healthcare level.

### Study participants and setting

The target population was healthcare professionals at selected primary healthcare clinics in KwaZulu-Natal.

Participants included mental healthcare nurses working in mental healthcare institutions, psychologists who have been seeing clients with depression and HIV, primary healthcare nurses, medical doctors working at mental healthcare institutions with mental health care users (MHCU), psychiatrists, healthcare professionals attending to clients in either primary healthcare services, HIV services and mental healthcare institutions, or those who have been working in these settings for a minimum of 2 years. Participants were expected to be knowledgeable and informative if they had at least two full years of uninterrupted service and experience within that particular setting. Psychiatrists and doctors, either working in mental healthcare institutions or who specialised in HIV, were also included. The purposive sampling method was employed to select the 200 healthcare professionals who participated in this study. The study was conducted in three selected healthcare facilities with HIV services, and mental healthcare facilities on site and in one mental healthcare institution, in KwaZulu-Natal. The three hospitals selected were at a district level. They were able to offer comprehensive HIV care as well as initiate and manage patients on antiretroviral therapy. Those hospitals were situated at eThekwini district in KwaZulu-Natal in the suburban area. The selected hospitals provided primary healthcare services and managed them accordingly. Patients using those facilities presented with several medical conditions, some of them being opportunistic infections to PLWH as well as mental health problems such as depression and anxiety.

### Data collection

A self-developed questionnaire was used to obtain information from the participants. The survey was designed to determine participants’ knowledge about the integration of mental healthcare services into HIV services. The Cronbach’s alpha value of the questionnaire was above 0.7% during the pilot process when the questionnaire was subjected to validation procedures. Second, it was given to different experts of mental health and HIV to critique and review it as part of the validation process of the questionnaire. Also, factor analysis was used to assess and identify each factor loading, that is, which item loaded underneath each component, and those items which did not load under each component were discarded.

The researcher obtained permission to conduct the study, prior to data collection, from the University of KwaZulu-Natal. Participants were requested to sign a declaration form as soon as they agreed to participate in the study. The researcher explained to them how to complete the survey to avoid wastage, errors and spoiled surveys. The participants were not coerced to participate. Confidentiality was maintained throughout the study.

### Measurements

The participants were asked to agree or disagree with a list of statements that related to their knowledge, on a five-point Likert scale (strongly disagree, disagree, neutral, agree and strongly agree). Content validity was used in the study to validate that the items within the instrument were measured for what they aimed to measure in terms of the research objectives.

Reliability was obtained by constructing questions of a simple manner to prevent misinterpretation and to construct different sections of the questionnaire in the same manner. The test–retest method was used in this study to assess the instrument’s stability over time (Patel et al. 2012). The reliability of the instrument was tested by administering the questionnaire to five registered nurses of the population and then administering the same questionnaire to the same participants 2 weeks later. The two rounds of the questionnaire from these respondents were then checked to see if the results would still be consistent, which would indicate the reliability of the questionnaire to elicit the necessary information as well as the internal consistency of the questionnaire.

### Data analysis

Data were captured on spreadsheets after having been gathered and examined for completeness. The surveys were coded, computed and analysed using the Statistical Package for Social Sciences (SPSS), Version 25.0.

Descriptive statistics were used to summarise and analyse data using frequency tables, as this provided an accurate and clearer picture of the results for easy understanding. One-way analysis of variance (ANOVA) followed up by Tukey’s honestly significant difference (HSD) was used for inferential statistics.

### Ethical consideration

Approval for this study was obtained from the Research Ethics Committee of the University of KwaZulu-Natal, Protocol number: HSS/2248/017D. The consent of participants was sought before they were enrolled in this study. The participants offered to take part in the study voluntarily (22 February 2018).

## Results

This section displays the demographic details as well as the knowledge of the participants about the integration of mental healthcare services into HIV services at a primary healthcare level.

### Demographic details

Of the participants, 32.5% (*n* = 65) were aged 26 to 35 years and 25% were aged 25 years or younger. A smaller portion, 17.5% (*n* = 35) were aged 36 to 45 years and about 25% (*n* = 50) were aged 46 years and above. The majority of the participants numbering 65% (*n* = 130) were women, while 35% (*n* = 70) were men.

Based on the number of years of experience of the participants, as healthcare professionals, working in mental healthcare, PHC and HIV services, the results show that 40% (*n* = 80) of the participants had 2 to 5 years of experience, while 22.5% (*n* = 45) of the participants had 6 to 10 years of experience, and 15% (*n* = 30) of the participants had 11 to 15 years of experience. A smaller number, 7.5% (*n* = 15), had 16 to 20 years of experience and a few had 20 or more years of experience.

Of the participants, 50% (*n* = 100) had 2 to 5 years of experience, 14.5% (*n* = 29) had 6 to 10 years of experience, 7.5% (*n* = 15) had 11 to 15 years of experience and 5% (*n* = 10) had 16 to 20 years of experience, in HIV services.

Of the participants, 32.5% (*n* = 65) had 2 to 5 years of experience, while 26% (*n* = 52) had 6 to 10 years of experience. A total of 14% (*n* = 28) of the participants had 11 to 15 years of experience, 22.5% (*n* = 45) had 16 to 20 years of experience and a mere 5% (*n* = 10) had 20 or more years of experience.

From the results obtained, the majority of the participants, 71% (*n* = 142), were mental health, primary healthcare and HIV-trained nurses, while 0.5% (*n* = 1) of the participants were psychiatrists and 21.5% (*n* = 43) of the participants were medical doctors. Only 5.5% (*n* = 11) of the participants were psychologists.

### Knowledge of participants about the integration of mental healthcare into HIV services at primary healthcare level

Table 1 shows that 91% (*n* = 182) of the participants had limited knowledge about the integration of mental healthcare services into HIV services at a primary healthcare level. Only 9% (*n* = 18) of the participants were knowledgeable about the integration of mental healthcare services into HIV services at a primary healthcare level.

**TABLE 1:** Knowledge of respondents about the integration of mental healthcare into HIV services at selected primary healthcare.

Knowledge score	Frequency	Percentage	Mean	Standard deviation
Knowledgeable	5	2.5	34.87	9.56
Not knowledgeable	182	91.0	34.87	9.56

Table 2 displays the participants’ knowledge about the integration of mental healthcare services into HIV services at a primary healthcare level. With regard to the statement ‘I have never received any form of formal education on mental health’, 42.5% (*n* = 85) agreed and stated that they have never received any form of formal education on mental health. But 42.5% (*n* = 85) disagreed and stated that they have received some form of formal education on mental health and 8.5% (*n* = 17) remained neutral on the statement.

**TABLE 2:** Participants’ responses on knowledge about the integration of mental healthcare into HIV services at selected primary healthcare.

Items	Strongly disagree		Disagree		Neutral		Agree		Strongly agree	
	*n*	%	*n*	%	*n*	%	*n*	%	*n*	%
I have never received any form of formal education on mental health.	68	34.0	30	15.0	17	8.5	55	27.5	30	15.0
I can identify a person who presents with mental health problems.	15	7.5	10	5.0	20	10.0	75	37.5	80	40.0
I can treat a person who presents with both mental health problems and HIV at a primary healthcare setting.	2	1.0	22	11.0	6	3.0	85	42.5	85	42.5
I do not have enough clinical skills to care for people with mental health problems and HIV.	40	20.0	45	22.5	20	10.0	55	27.5	40	20.0
I do not have enough clinical skills to care for people with mental health problems.	40	20.0	35	17.5	0	10	75	37.5	30	15.0
I provide information about mental health to patients.	10	5.0	25	12.5	5	2.5	65	32.5	95	47.5
I assess my patients for physical problems related to psychotropic drugs use.	10	5.0	20	10.0	5	2.5	85	42.5	80	40.0
There are guidelines available for the implementation of the national mental health policy framework.	10	5.0	35	17.5	10	5.0	85	42.5	60	30.0
Guidelines on the implementation of national mental health policy framework were read and explained using simple language to me.	25	12.5	15	7.5	15	7.5	85	42.5	60	30.0
I was given an opportunity to seek for clarity on the guidelines or the available document of the implementation of the national mental health policy framework.	45	22.5	35	17.5	20	10.0	45	22.5	55	27.5
District personnel visited us to provide guidance on the available document of the implementation of the national mental health policy framework on the integration of mental health care into HIV services.	30	15.0	50	25.0	35	17.5	60	30.0	25	12.5
Workshops or in-service education was conducted for the employees regarding the implementation of the national mental health policy framework.	25	12.5	40	20.0	55	27.5	50	25.0	30	15.0
Testing and evaluation of the national mental health policy framework was done by the district and immediate supervisors.	45	22.5	45	22.5	35	17.5	50	25.0	25	12.5
There is a need to implement the national mental policy framework on the integration of mental healthcare into HIV services.	10	5.0	20	10.0	10	5.0	80	40.0	80	40.0

The results obtained from the responses of the participants to the statement ‘I can identify a person who presents with mental health problems’ show that a good number of the participants, 77.5% (*n* = 155), agreed that they can identify a person presenting with mental health problems. A total of 10% (*n* = 20) of the participants remained neutral, while 12.5% (*n* = 25) of the participants disagreed.

The survey also sought to determine the view of healthcare professionals on the statement ‘I can treat a person who presents with both mental health problems and HIV in a primary healthcare setting’. Majority of the participants were of the view that a person who presents with both mental health problems and HIV can be treated in a primary healthcare setting. A total of 85% (*n* = 170) of the participants agreed. However, 11.5% (*n* = 23) of the participants disagreed and 3% (*n* = 7) of the participants refrained from answering the question.

Assessing the clinical skills of the healthcare professionals’ abilities to care for people with mental health problems and HIV, it was asked whether they thought that they had the required skills to care for such patients. Results showed that 47.5% (*n* = 95) of the participants agreed. However, 42.5% (*n* = 85) of the participants disagreed that they had the required skills to care for persons with HIV and mental health problems. A total of 10% (*n* = 20) of the participants remained neutral and did not respond to the statement.

Regarding the statement ‘I do have the required clinical skills to care for people with mental health problems’, most of the participants agreed that they have the required skills to manage patients with mental health problems. A total of 52.5% (*n* = 105) of the participants agreed. However, 45% (*n* = 90) of the participants disagreed that they possess the skills needed to care for people with mental health problems, while 2.5% (*n* = 5) remained neutral on the statement.

It was observed, from the responses of the participants, that they provide patients with the necessary information about mental health. Of the participants, 80% (*n* = 160) agreed that they provide patients with information on mental health. A minority of the participants refrained from providing a response to this statement, while 17.5% (*n* = 35) disagreed, thereby stating that they do not provide information to patients about mental health.

On the issue of guidelines available for the implementation of a national mental health policy framework, 72.5% (*n* = 145) of the participants agreed that there are guidelines available for the implementation of a national mental health policy framework. Although the majority of the participants are in favour of availability of guidelines needed for the implementation of a national mental health policy framework, 15% (*n* = 30) of the participants disagreed that there are frameworks in existence that are needed to implement a national mental health policy. A total of 5% (*n* = 10) of the participants remained neutral on the statement.

The responses of the participants to the statement ‘guidelines on the implementation of a national mental health policy framework were read and explained to me using simple language’ show that most of the participants accept that the guidelines on the implementation of a national mental health policy framework were read and explained to them. A total of 72.5% (*n* = 145) of the participants agreed. Nevertheless, 20% (*n* = 40) of the participants disagreed, while 7.5% (*n* = 15) of the participants remained neutral on the statement.

The participants’ responses to the question ‘I was given the opportunity to seek clarity on the guidelines or the available document for the implementation of a national mental health policy framework’ show that 50% (*n* = 100) agreed, 40% (*n* = 80) disagreed and 10% (*n* = 20) refrained from responding to the statement.

The participants were asked if the district personnel visited their facility to provide guidance on the available documentation for the implementation of a national mental health policy framework on the integration of mental healthcare services into HIV services. From the participants’ responses, it was gathered that 42.5% (*n* = 85) agreed that the district personnel had visited in order to provide guidance on the available documentation for the implementation of a mental policy framework. However, 40% (*n* = 80) of the participants disagreed, stating that the district personnel have not visited to provide guidance on a mental health policy framework implementation.

With regard to the organisation of workshops or in-service education to enlighten the participants about the implementation of a national mental health policy framework, 27.5% (*n* = 55) remained neutral and did not respond to the statement. A total of 40% (*n* = 80) agreed that workshops or in-service training are conducted by employers to enlighten employees on the implementation of a national mental health policy framework. A total of 32.5% (*n* = 65) disagreed, stating that there were no workshops or in-service training in place to assist health professionals with the implementation of a national mental health policy framework.

The healthcare professionals, in this study, were asked if the district and immediate supervisors did test and evaluations of the national mental health policy framework. The results obtained showed that there was almost a balance between the participants who responded positively and negatively. A total of 37.5% (*n* = 75) agreed, 17.5% (*n* = 35) of the participants abstained from responding and 40% (*n* = 80) disagreed that testing and evaluation of the national mental health policy framework was done by the district and immediate supervisors.

Lastly, the participants were asked if there is a need to implement a national mental health policy framework on the integration of mental healthcare services into HIV services and the majority of the healthcare practitioners believe that there is a need to implement a national policy framework that integrates mental healthcare services into HIV services. A total of 80% (*n* = 160) agreed that there was a need for the integration of mental healthcare services into HIV services, while 20% (*n* = 40) disagreed.

### Mean differences

When a one-way ANOVA test was run to determine mean differences in knowledge scores between mental healthcare practitioners of different years of service, results showed that there were statistically significant differences between groups, with a *p* value of 0.000 (*p* = 0.00). The one-way ANOVA was then followed-up by running a Tukey’s HSD post hoc test, also known as Tukey, as our data met the homogeneity of variances assumption. The aim was to control the experiment wise error rate (usually alpha = 0.05). Post hoc comparisons using the Tukey’s HSD test indicated that the mean score of knowledge for the MHCPs with service number of years 2–5 and 20 years and above (*p* = 0.00) was significantly different than mean difference between 2–5 and 16–20 (*p* = 0.001). However, there were no differences between the groups of 2–5 and 16–20 years of service (*p* = 0.992). The results revealed that knowledge of participants from each facility indicate that there was no statistically significant difference among the three facilities (*p* = 0.757). However, even though the results were not significantly different, we noticed that hospitals A and C had slightly higher mean knowledge scores when compared to hospital B as shown in the descriptive table on the results.

## Discussion

In this study, results obtained show that the majority of the healthcare professionals, 91%, had limited knowledge regarding the integration of mental healthcare services into HIV services at a primary healthcare level. Very few, a mere 9%, had adequate knowledge about the integration of mental healthcare services into HIV services. In hospital settings where patients infected with HIV are managed, it is common to find different healthcare professionals working together as a team (Joska & Sorsdahl 2012). There is a need to promote for the integration of mental healthcare services into HIV services at a primary healthcare level, as there is a high incidence of HIV infection in South Africa and there is a possibility that this infection occurs mostly in patients suffering from severe mental ailments (Joska, Kaliski & Benatar 2008). Healthcare professionals at this level need to be adequately trained and up to date with modern trends to effectively diagnose and manage mental disorders in HIV-infected patients (Joska & Sorsdahl 2012).

Results show that an equal number of participants either received some form of formal education on mental health or received none at all. Healthcare professionals are responsible for providing care as well as accurate information to patients and their relatives regarding the condition of their patients. It is imperative that these caregivers have adequate knowledge about the disease. This is vital for the delivery of the best possible service to meet their patient’s healthcare need (Umeh et al. 2008). It is essential to put in place the necessary structures to conduct in-service training as well as pre-service training on mental healthcare, for healthcare professionals at a primary healthcare level, to boost productivity and quality of service (Hanlon et al. 2014).

It was observed in this study that a good number of the participants, 77.5% to be exact, could identify a person who presents with mental health problems. This was encouraging. Studies conducted by Modula and Ramukumba (2018) on nurses show that there are observable flaws in the diagnosis of mental ailments at the primary healthcare level. They went on to state that this may result from a deficiency of the required skills and inadequate facilities for screening (Modula & Ramukumba 2018). It is essential for policy-makers in education to develop teaching approaches that will enable and promote the acquisition of the needed interpersonal and assessment competencies in nursing (Modula & Ramukumba 2018).

This study has also shown that the majority (85%) of the participants are of the view that a person who presents with both mental health problems and HIV can be treated at a primary healthcare level. This belief is also held by most healthcare professionals who are in support of an efficient primary healthcare system that takes into account both physical and behavioural medical disorders (Crowley & Kirschner 2015). It was also observed in this study that close to an average number (47.5%) of the participants believed that they had the skills needed to care for patients suffering from HIV and mental problems, while 42.5% thought otherwise. A study by Maconick et al. (2018) showed that some of the primary healthcare nurses, in their study, felt that they had little to offer their patients with regard to mental health challenges and this, in turn, affected their productivity level. This can be reversed if the primary healthcare nurses are competent in handling the mental challenges of their patients (Maconick et al. 2018). The more competent these healthcare professionals are, the better their position will be to disseminate information to their patients. This study also found that most of the healthcare professionals (80%) in the study provide their patients with medical information regarding mental health.

The study also sought to determine if healthcare professionals examine patients for physical conditions that are associated with the use of psychotropic drugs. It was observed that most of the healthcare professionals (82.5%) assess patients for physical problems that are related to the use of psychotropic drugs. It is important to assess HIV infected persons for physical problems because of the high level of depression among HIV-infected persons and because the use of substances may impact the patient’s adherence to antiretroviral treatment (Skalski et al. 2015). Studies have shown that there is an increase in mental disorders associated with substance use and among patients infected with HIV (Chander, Himelhoch & Moore 2006; DeLorenze et al. 2014; Skalski et al. 2015).

A good number (72.5%) of the participants in this study agreed that there are guidelines available for the implementation of a national mental health policy framework and that these guidelines have been read and explained to them using simple language. There are mental health guidelines in existence for the management of patients infected with HIV in South Africa (Johnsson 2013). These guidelines are anticipated to help improve the knowledge and capacity of primary healthcare HIV clinicians in dealing with mental health challenges. This will also help to increase the awareness of healthcare professionals on the importance of integrating HIV and mental healthcare into routine practice (Johnsson 2013).

There were conflicting reports by participants as to whether they were given the opportunity to seek clarity on the guidelines or available documentation for the implementation of a national mental health policy framework. There is a need for clarity on the guidelines to enable healthcare professionals to utilise it properly as a tool to manage HIV-infected patients efficiently. This will enable healthcare professionals to have all the required information on the evaluation and initiation of treatment (Modula & Ramukumba 2018). To assist healthcare professionals in gaining a better understanding on the implementation of the guidelines, it is important for district personnel to visit healthcare facilities to provide guidance on implementation of the national mental health policy on the integration of mental healthcare services into HIV services. However, it was observed from the participants’ responses that the visits of district officers were not as they should be, 42.5% of respondents reported that visits were taking place.

Research has shown that the implementation of mental healthcare into services at the level of primary healthcare can be achieved by employing in-service training (Liu et al. 2016; Maconick et al. 2018). This study discovered that the participants’ responses to the organisation of workshops or in-service training were inadequate, as there was almost a balance between those who agreed and those who disagreed regarding the need to organise programs that will enlighten employees on implementation of a national mental health policy framework. A study has found that engaging the staff of a clinic in long-term workshops and in-service training programs has significant benefits for the integration of mental healthcare into services at a primary healthcare level (Maconick et al. 2018).

Finally, the majority (80%) of the participants believe that it is of great importance to implement a national policy framework that integrates mental healthcare services into HIV services. The integration of these services into one clinic will help to reduce costs and increase the efficiency in the management of patients that present with these conditions (DeLorenze et al. 2014).

## Limitation

This study was conducted in selected healthcare facilities in KwaZulu-Natal, South Africa, hence the need for a broader study to be done in order to obtain a bigger picture of the problem in question.

## Implications for future research

A study on healthcare policy-makers and administrators’ opinions on the implementation of integrating mental healthcare services into HIV services should be conducted. This will help to properly understand and establish the readiness of all stakeholders to integrate mental healthcare services into HIV services at a primary healthcare level.

## Recommendation

Programmes should be put in place to educate and train healthcare professionals on the need to examine and promote appropriate care, treatment and support. The administrators responsible for the implementation of the national policy framework should enhance its speedy implementation because of the great benefit it brings.

## Conclusion

This study aimed at exploring the knowledge of healthcare professionals regarding the integration of mental healthcare services into HIV services at a primary healthcare level. It was found that most of the participants had limited knowledge about the subject matter. This, therefore, shows that all stakeholders involved need to implement initiatives to address this knowledge gap.
